# Multi-point collaborative mobile replica node detection protocol based on key negotiation

**DOI:** 10.1038/s41598-026-44298-7

**Published:** 2026-03-23

**Authors:** Jun Cheng, Zhiming Zhang, Jinfeng Li

**Affiliations:** 1 College of Aeronautical Information Engineering, Jiangxi Flight University, Nanchang, 330088 Jiangxi China; 2 School of Artificial Intelligence, Jiangxi Normal University, Nanchang, 330027 Jiangxi China

**Keywords:** Replica node detection, Mobile wireless sensor networks, Detection rate, Storage overhead, Communication overhead, Network lifetime., Engineering, Mathematics and computing

## Abstract

Replica node attacks, a common issue in wireless sensor networks (WSNs), can cause major damage. Most traditional replica node detection protocols are designed for static WSNs and are limited by easy information leakage, high storage and communication overhead, and a short network lifetime. Therefore, this study proposes a multi-point collaborative mobile replica node detection protocol based on key negotiation, referred to as KN-MCDP. The KN-MCDP scheme is designed for use in mobile wireless sensor networks (MWSNs) where a limited number of mobile nodes are deployed in static WSNs. The protocol can not only identify replica nodes in static WSNs but also determine whether a mobile node is a replica, thereby providing enhanced network protection. When cluster head nodes and mobile nodes communicate, they encrypt the exchanged information using digital signature technology, ternary symmetric polynomial technology, and symmetric encryption technology, thus preventing information leakage collected by cluster head nodes. Collecting network information using Bloom filters on cluster head nodes and mobile nodes reduces network storage and communication overhead. In different phases, the protocol employs cluster head nodes, mobile nodes, and the base station to identify and isolate replica nodes. This approach balances the energy overhead of the network and extends its lifetime. The experimental results demonstrate that the KN-MCDP protocol can achieve a high detection rate, reduce the network’s storage and communication overhead, balance energy overhead, and extend the network lifetime.

## Introduction

The Internet of Things (IoT) industry is growing rapidly. The global IoT market and its application areas continue to expand, and governments have implemented policies to support research and development (R&D) efforts^1–3^. The core technologies of IoT include sensor technology^4^, network communication technology, radio frequency identification (RFID)^5^, and data processing technology^6^. As a crucial technology in IoT, wireless sensor networks connect the information world to the human world, making it easier for people to access information. However, due to the open deployment area and limited node resources, sensor nodes are susceptible to various attacks^7^. For instance, the replica node attack, in which an attacker captures a sensor node and duplicates many nodes with the same identity document (ID) as the captured node by analyzing critical information such as node ID and secret keys, is common. This information can be used to cause further damage to the network^8^. Since the attack originates within the network^9,10^, it is difficult to detect and is more destructive. By using replica nodes, the adversary can forge or tamper with routing information, causing network routing chaos or fragmentation^11,12^. Likewise, the adversary can send false target information, leading to data fusion errors^13^. Additionally, the adversary can disrupt the network’s time synchronization, leading to larger synchronization errors in the system^14,15^. For traditional WSNs, replica node attacks are extremely damaging. Hence, researching effective replica node detection protocols to identify and isolate these nodes in the network is a critical issue, especially in the field of information security.

Numerous studies have been conducted, and various replica node detection protocols have been proposed to detect and isolate replica nodes in WSNs. Traditional protocols designed for static WSNs have issues such as leakage of cluster head node information, high storage and communication overhead, and unbalanced energy consumption. Therefore, the KN-MCDP scheme is proposed for a mobile wireless sensor network in which a limited number of mobile nodes are deployed within static WSNs. The scheme comprises the intra-cluster localized detection phase, the communication phase between mobile nodes and intra-cluster nodes, and the mobile inter-node communication phase. Compared to traditional replica node detection protocols, the innovations of the KN-MCDP protocol are as follows:

1. During the communication phase between mobile nodes and intra-cluster nodes, the communicating parties encrypt the exchanged information using digital signatures, ternary symmetric polynomial technology, and symmetric encryption technology. This helps prevent information leakage from the cluster head.
2. Cluster head nodes gather information from intra-cluster nodes using Bloom filters, and mobile nodes collect network-wide data with Bloom filters. Using the high storage space efficiency of Bloom filters reduces the storage overhead of the network. Since the Bloom filter itself is small, the communication overhead required to transmit it is also reduced, thereby decreasing the network communication overhead.
3. The protocol can not only identify replica nodes in static networks but also determine whether a mobile node is a replica node. During communication between mobile nodes and intra-cluster nodes, the cluster head nodes and the selected cluster member nodes can determine whether a mobile node is a replica node by checking whether a relevant parameter exceeds a threshold value. In the mobile inter-node communication phase, if two mobile nodes interacting with each other have the same ID, it can be determined that the mobile node with this ID is a replica node.
4. The energy overhead of the network is balanced by using the cluster head node to identify replica nodes within the cluster, several high-energy mobile nodes to identify replica nodes across the network, and the base station to identify mobile nodes as replica nodes. This approach extends the network’s lifetime.

The remainder of the paper is organized as follows. Section "Related works" reviews related works. Section "System model and prerequisite knowledge" outlines the system model of the KN-MCDP protocol. Section "KN-MCDP protocol" details the specific steps of the KN-MCDP protocol. Section "KN-MCDP protocol analysis" examines the security and the different overheads of the protocol. Section "Experimental results" presents the results of the protocol experiments. Finally, the conclusion is presented in Section "Conclusions".

## Related works

Currently, many replica node detection protocols have been proposed. These protocols can be broadly classified into two categories^16^: centralized replica node detection protocols and distributed replica node detection protocols.

Centralized replica node detection is simple. Parno et al. introduced a straightforward centralized detection protocol, referred to as the Base Station (BS) protocol^17^. In this protocol, each node transmits its neighbor and location information to the base station, allowing the base station to easily identify nodes with the same ID in different locations. The BS protocol can achieve a high detection rate. However, as the number of nodes in the network increases, communication and storage overhead near the base station surges dramatically, inevitably affecting the network lifetime. Consequently, this approach presents significant challenges in scalability, energy consumption, network lifetime, and related metrics. Later, Xing proposed a fingerprint authentication detection program^18^ to improve the BS protocol and reduce some communication and storage overhead. Despite these improvements, centralized replica node detection methods remain limited by single-point failure and network load imbalance^19^. Therefore, this study proposes a distributed replica node attack detection scheme suitable for WSNs.

Distributed replica node detection protocols can mainly be conceptualized as follows: Sensing nodes initially broadcast messages indicating their locations, and the system periodically monitors the network to determine whether different geographic locations correspond to the same ID^20^. Based on the BS protocol, Parno et al. introduced the Random Multicast (RM) detection protocol. In RM, sensor nodes in the network periodically send their position information to a randomly chosen set of witnesses. Replica nodes can be identified using the birthday paradox. Zhu et al. proposed the Line-Selected Multicast (LM) protocol^21^, which differs from the RM protocol in that its witness nodes are selected from a small, restricted region rather than the entire network, thereby enhancing the detection rate. Li et al. proposed the Distributed Hash Table (DHT) protocol^22^, which is a fully decentralized, key-based caching and verification system that can effectively detect cloned nodes. The DHT protocol offers higher storage efficiency and security than the LM protocol. The detection protocols introduced by Devi ^23^ and Vaishnavi^24^ achieve higher detection rates and reduce communication overhead.

In China, H. Zhou et al. proposed the Cluster-Based Detection Method (CBDM) protocol^25^, which uses cluster heads for local detection. Each cluster head then transmits data to the base station for global detection. This local-to-global method addresses the issue of high communication overhead between the base station and nearby nodes in centralized schemes. Later, J. Cheng improved the CBDM protocol and introduced the Bloom Filter-Based Cluster Detection Protocol (BFCP)^26^. Cluster head nodes no longer use traditional storage methods. Instead, each cluster head has a Bloom filter to store relevant data, thereby reducing storage requirements and extending the network lifetime. However, if no replica nodes are detected during the intra-cluster detection phase, the cluster head node will gather information from all other cluster head nodes to identify them, increasing communication and storage overhead for the cluster head nodes.

Previous detection protocols are better suited to static WSNs, whereas replica node detection protocols for mobile wireless sensor networks are rarely studied. MWSNs are also a type of wireless sensor network, and the mobility of the sensor nodes is the most critical feature. MWSNs mainly comprise two categories^27^: (1) networks where all nodes are mobile, and (2) a heterogeneous hybrid network formed by deploying some mobile nodes in a static WSN. Because the nodes in MWSNs are mobile, they face more security threats and more complex issues than those in static networks.

Furthermore, Anthoniraj proposed Cluster-Based Cloning Attack Detection (CBCD)^28^ to identify cloned nodes in MWSNs. This protocol divides the sensor network into multiple clusters and encrypts communication between cluster heads and the base station using a symmetric key. It uses a secret-key negotiation method to rapidly detect clone nodes. Nevertheless, the cluster head node collects information from nodes within the cluster using conventional methods, resulting in significant storage overhead for the cluster head node. Additionally, the protocol uses only pre-shared symmetric keys for communication encryption, which limits its security mechanism. M. Sajitha introduced a new Whale-based Node Identity Verification (WbNIV)^29^, which uses the whales’ fitness function to determine energy levels. Nodes in the network are tracked, and their energy levels are measured. Since replica nodes have lower energy levels than normal nodes, they can be easily detected by their energy levels. However, the security of WbNIV relies on the heuristic assumption that "replica nodes consume less energy than normal nodes." Attackers can easily circumvent replica node detection by using devices with sufficient energy. The protocol attempts to address a cryptographic security issue by relying on an unreliable and evadable energy characteristic, casting doubt on its inherent reliability. Sujihelen proposed a Strategic Security System (SSS)^30^ to detect replica nodes in static and dynamic distributed WSNs. The protocol uses Single Stage Memory Random Walk with Network Division (SSRWND) and Random Walk-based (RAWL) approaches. RAWL targets dynamically distributed WSNs, while SSRWND predicts, detects, and isolates replicated nodes in static networks. The system uses less memory and offers higher detection accuracy, lower time delay, and reduced communication overhead. It should be noted that memory audits and network segmentation based on random walks rely on network topology and probability theory, and thus their detection rates are constrained by these models. Simultaneously, SSS requires the deployment and maintenance of two sets of detection logic, increasing protocol overhead and reducing network lifetime. In summary, existing mobile replica node detection protocols each have their own shortcomings in detection rate, security, and overhead. There is an urgent need to propose a mobile replica node detection protocol that comprehensively considers detection rate, security, overhead, and network lifetime.

Inspired by relevant literature, this study proposes the KN-MCDP scheme for a mobile wireless sensor network with a limited number of mobile nodes deployed within static WSNs. KN-MCDP is not merely a simple patch or parameter adjustment to existing solutions, but rather a substantive new approach proposed at the levels of design philosophy, technology integration, and system architecture.

Multi-level security settings ensure the safety of data transmission by cluster head nodes; employing Bloom filters to store and compress information reduces network storage and communication overhead; and using cluster head nodes, high-energy mobile nodes, and the base station for replica node detection and isolation balances network costs while extending network lifetime. Simultaneously, expanding the scope of replica node detection to include mobile nodes themselves enhances protocol security and improves detection rates. Collectively, these innovations ensure cluster head data security, increase the detection rate, reduce storage and communication overhead, balance energy consumption, and extend the network lifetime.

## System model and prerequisite knowledge

### Network and attack model

#### Network model

It is assumed that the wireless sensor network consists of static sensor nodes and multiple mobile nodes.

Static sensor nodes form clusters using a designated algorithm^31^. Each cluster includes a cluster head node and cluster member nodes. The cluster head node knows the cluster number to which it belongs. Each cluster head node creates two m-bit Bloom filters and a ternary symmetric polynomial. Cluster member nodes send their IDs and location data to the cluster head node. The cluster head node uses Bloom filters to store the corresponding information.

The mobile node moves through the network following the Random Direction model^32^. It carries two Bloom filters of M bits. The mobile node can communicate only with other mobile nodes and the cluster head node that carries the Bloom filter. All communications are encrypted and signed using identity-based public key cryptography schemes^33,34^.

#### Attack model

Assuming that replica node attacks are categorized into standard attacks and enhanced attacks.

Under standard attack conditions, suppose the attacker captures one or more nodes and extracts private information. This data allows them to create multiple replica nodes with the same ID and valid private credentials. These replica nodes can establish trust with other nodes, monitor network traffic, steal data, and inject false information into the network, posing a serious threat.

In enhanced attack scenarios, attackers may conduct side-channel attacks^35^ by analyzing a node’s power consumption, electromagnetic emissions, or timing information to infer intermediate states or key information related to polynomial computations within the KN-MCDP protocol. Attackers may perform partial node memory scans or fault injection attacks^36^, obtaining partial confidential information without completely destroying the node.

### Random direction model

Assume all nodes are distributed in a two-dimensional region $U$ with area $D$, and each node has a transmission radius $R$. In the Random Direction model, one epoch of the mobile node follows this process^37^: (i) randomly select a direction $\theta$ from $[0,2\pi )$; (ii) randomly select a speed from a predefined range $[v_{\min } ,v_{\max } ]$, and the average speed is $\mathop v\limits^{\_}$; (iii) select a continuous moving duration $T$ with an average value of $\mathop T\limits^{ - }$, such that the average moving distance in $T$ time is $\mathop L\limits^{ - } = O(\sqrt D )$ with a value of $\mathop T\limits^{\_} \times \mathop v\limits^{\_}$; (iv) move in the direction $\theta$ at speed $v$ for duration $\mathop T\limits^{{}}$; (v) pause for a random duration $T_{stop}$; and (vi) repeat from step (i).

The expected encounter time between two mobile nodes is the average time required for them to move within each other’s transmission range within the deployment area $U$ of the sensor network. This is illustrated in Eq. (1):

1 $$ET_{m} (RW) = \frac{1}{2}N(0.34 \cdot \log (D) - \frac{{2^{R + 1} - R - 2}}{{2^{R} - 1}})$$

Where $ET_{m} (RW)$ denotes the node’s expected encounter time.

The Random Direction model is a widely adopted theoretical framework for mobile self-organizing networks and mobile wireless sensor networks. It describes the randomness and memorylessness of node movement through simple parameters (speed, direction, pause time), providing a fair, repeatable, and analyzable benchmark for comparing mobility-related performance across different protocols. In many large-scale, long-term monitoring tasks, the specific path of an individual node may be constrained by local conditions. However, from the perspective of the network’s overall statistical characteristics, its macro-level effect is comparable to that of the Random Direction model. The performance of our proposed KN-MCDP protocol relies strongly on this long-term statistical coverage property.

As we will see below, key parameters of the KN-MCDP protocol, such as the number of mobile nodes and their movement speed, are adjustable. In practical deployments, if environmental constraints are known, we can compensate for uneven coverage by appropriately increasing the number of mobile nodes, thereby maintaining the expected detection efficiency.

### Prerequisite knowledge for key technologies

#### Ternary symmetric polynomial

In this study, we used a ternary symmetric polynomial to bind node information. The ternary symmetric polynomial is shown in Eq. (2), where the coefficient $b_{ijk}$ belongs to the finite field $F_{q}$ and $q$ is a sufficiently large prime number^38^.

2 $${\mathrm{f}}_{1} {\mathrm{(x,y,z)}} = \sum\limits_{{i,j,{\mathrm{k}} = 0}}^{t} {b_{{ij{\mathrm{k}}}} } x^{i} y^{j} z^{k} \, (b_{ijk} \in F_{q} )$$

The ternary symmetric polynomial has symmetry^39,40^. The symmetry of a ternary symmetric polynomial refers to the fact that when the positions of the three variables are exchanged arbitrarily, the value of the resulting polynomial does not change, e.g., ${\mathrm{f}}_{1} {\mathrm{(x,y,z)}} = {\mathrm{f}}_{1} {\mathrm{(z,y,x)}} = {\mathrm{f}}_{1} {\mathrm{(y,z,x)}}$. By leveraging the security of this polynomial, node information can be incorporated into the polynomial to modify its coefficients, thereby binding the node information to the polynomial. Using the symmetry of this polynomial, the authenticity of the coefficients bound to it can be verified, thus verifying the identity of the node.

#### Bloom filter

Burton Howard Bloom proposed the Bloom filter, a data structure for element insertion and query operations. Compared to other data structures, it is widely used for its efficient query time and space efficiency^41^. Here, we explain Bloom filter insertion and query operations.

It is assumed that the Bloom filter has a sequence of M addresses, and it uses k mutually independent hash functions to implement the insertion of a set of n elements $$S$$$$= \{ x_{i} \}$$$$(i = 1,2...n)$$.

**Fig. 1:**
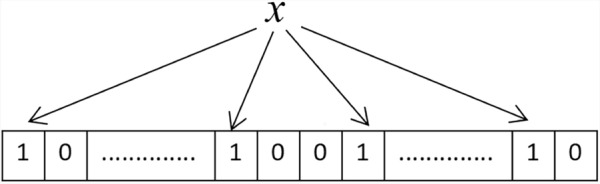
Schematic diagram of a bloom filter.

As depicted in Fig. 1, initially, all address sequences in the Bloom filter are set to 0. To insert a certain element $x \in S$, the Bloom filter applies k mutually independent hash functions to compute mappings to k corresponding address sequences, setting the corresponding positions to 1 to complete the insertion process.

When checking if an element is stored in the Bloom filter, the same k hash functions are used. If the address sequence of all k mappings is 1, it suggests that the element may be in the set. Otherwise, the element is not present.

Notably, since the Bloom filter’s address sequence has only m bits, when a large number of elements are inserted, some address bits may already be set to 1. This can cause an element outside the set to be mistakenly recognized as a member, leading to a false-positive error^42,43^. False-positive errors cause false alarms in Bloom filters. Specifically, the approximate formula for the false alarm rate of the Bloom filter is as follows:

3 $$p \approx (1 - e^{ - kn/M} )^{k}$$

Here, p represents the false alarm rate. With the simulation parameters M = 700, k = 5, and n = 100, we obtain p $\approx 3.46\%$, which is acceptable in wireless sensor networks. Furthermore, because the network comprises multiple mobile nodes, a false positive from the Bloom filter at one node can be compensated for by others, thereby mitigating the overall false alarm rate across the network.

#### Security protocols and their functions

The KN-MCDP protocol employs three security mechanisms: digital signatures, a ternary symmetric polynomial, and symmetric encryption. The digital signature provides identity authentication and enables non-repudiation between communicating parties; the ternary symmetric polynomial supports lightweight key predistribution and session negotiation while offering security resilience against node capture attacks; the symmetric encryption provides efficient confidentiality protection for the actual transmitted business data.

In our solution, the aforementioned three security mechanisms form a layered, collaborative defense system. First, digital signature technology provides non-repudiation assurance for interactions between mobile nodes and cluster head nodes, as well as among mobile nodes themselves, ensuring the trusted origin of data transmission. After completing identity authentication, the system uses pre-stored ternary symmetric polynomials in mobile nodes and cluster head nodes—bound to node identity information—to generate session keys between nodes. Simultaneously, it leverages the mathematical properties of these polynomials to enhance the system’s resistance to replica node attacks. Finally, based on the shared session key derived from ternary symmetric polynomials, efficient symmetric encryption is applied to data exchanged between nodes, thereby ensuring the confidentiality of the data transmission process.

## KN-MCDP protocol

The KN-MCDP scheme includes three phases: the intra-cluster local detection phase, the communication phase between mobile nodes and intra-cluster nodes, and the mobile inter-node communication phase.

### Intra-cluster local detection phase

**Fig. 2:**
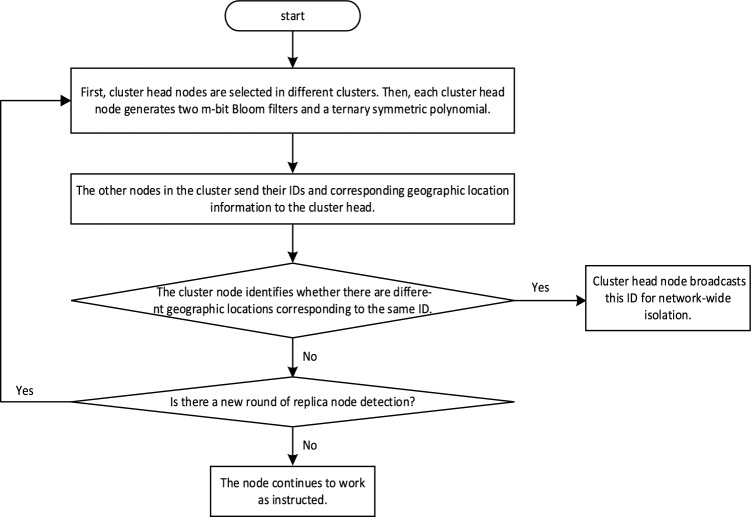
The intra-cluster local detection phase of the KN-MCDP.

During each detection cycle, cluster head nodes are selected probabilistically. Suppose there are i cluster head nodes, i.e., $$CH_{1} ,CH_{2} ,......CH_{i}$$ are selected. Each cluster head generates two m-bit Bloom filters: $$B_{ID} (CH_{i} )$$ and $$B_{l} (CH_{i} )$$, and creates a ternary symmetric polynomial: $$f_{1} (x,y,z) =$$$$\sum {a_{ijk} } x^{i} y^{j} z^{k}$$$$(0 \le {\mathrm{i}},j,k \le t)$$. Subsequently, the cluster head node broadcasts its status. Other nodes join the cluster with the lowest communication cost and send their IDs and geographic location information to the cluster head node. The cluster head stores new IDs into $$B_{ID} (CH_{i} )$$ and corresponding geographic location information into $$B_{l} (CH_{i} )$$. If the sent ID is not in $$B_{ID} (CH_{i} )$$, it is saved by $$B_{ID} (CH_{i} )$$, and the geographic location information corresponding to the ID is saved by $$B_{l} (CH_{i} )$$. If the sent ID is in $$B_{ID} (CH_{i} )$$ and the geographic location information does not exist in $$B_{l} (CH_{i} )$$, the node is judged as a replica node, and an error message is broadcast for network-wide isolation. The flowchart for the intra-cluster local detection phase of the KN-MCDP scheme is shown in Fig. 2.

### Communication phase between mobile nodes and intra-cluster nodes

**Fig. 3:**
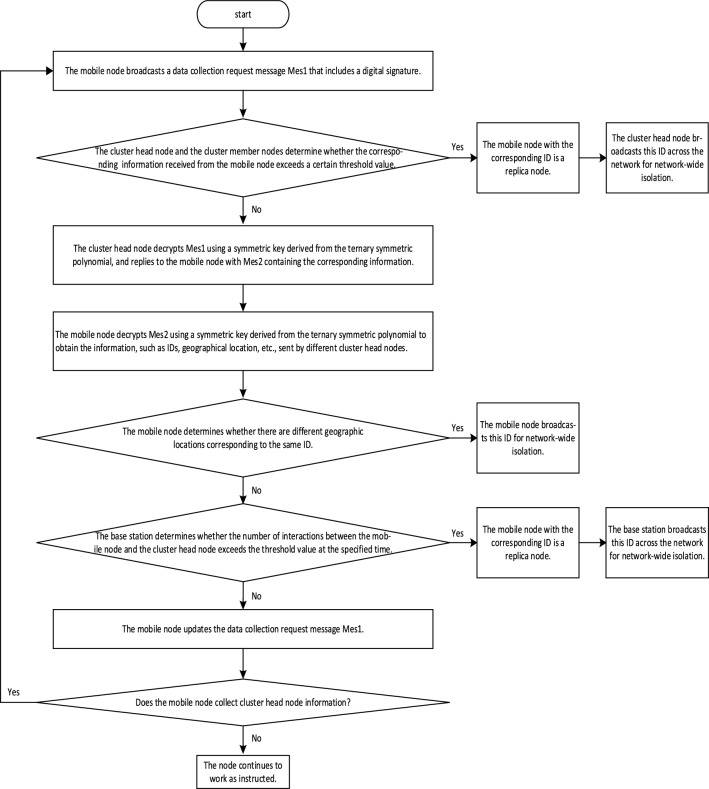
The overall flowchart for the communication phase between mobile nodes and

After the intra-cluster local detection phase, it proceeds to the communication phase between mobile nodes and intra-cluster nodes. The overall flowchart illustrating the communication phase between mobile nodes and intra-cluster nodes in the KN-MCDP scheme is shown in Fig. 3.

intra-cluster nodes of the KN-MCDP.

(1) After performing the judgment of replica nodes within the cluster, the mobile nodes enter the network. Every mobile node generates two M-bit Bloom filters: $B_{{ID_{mob} }}$ and $B_{{l_{mob} }}$, and creates a ternary symmetric polynomial: $f_{1} (x,y,z) = \sum {a_{ijk} } x^{i} y^{j} z^{k} (0 \le {\mathrm{i}},j,k \le t)$. Here, $B_{{ID_{mob} }}$ and $B_{{l_{mob} }}$ are used to collect the ID and the geographic location information, respectively. The ternary symmetric polynomial is used for communicating with the cluster heads.

Every mobile node moves randomly through the network based on the Random Direction model. It broadcasts a data collection request message $Mes_{{_{1} }}$ with its digital signature, and the content format is denoted as:

4 $${\mathrm{M}}es_{1} = \{ ID_{mob} ,t_{{arrive_{{\mathrm{i}}} }}^{mob} ,Count,SIG_{{{\mathrm{SK}}_{{{\mathrm{ID}}_{{{\mathrm{mo}}b}} }} }} (({\mathrm{Re}} q,T_{m} )||ID_{mob} )\}$$

Where $ID_{mob}$ denotes the identifier document of the mobile node; $t_{{arrive_{i} }}^{mob}$ indicates the time when the cluster head node $CH_{i}$ detects the arrival of the mobile node $mob$ in the cluster ${\mathrm{C}}_{i}$; $Count$ corresponds to the number of times the mobile node $mob$ has exchanged information with the cluster head nodes in the network; $SIG_{{{\mathrm{SK}}_{{{\mathrm{ID}}_{{{\mathrm{mo}}b}} }} }} (({\mathrm{Re}} q,T_{m} )||ID_{mob} )$ indicates that $({\mathrm{Re}} q,T_{m} )$ and $ID_{mob}$ are digitally signed by the private key of the $mob$; $T_{m}$ signifies the replica node detection cycle number stored by the $mob$; ${\mathrm{Re}} q$ denotes the data collection request sent by the $mob$.

(2) When a mobile $mob$ node initially enters the cluster $C_{i}$ and is within the communication radius of the cluster head node $CH_{i}$, $t_{{arrive_{i} }}^{mob}$ and $Count$ are stored by the $CH_{i}$. To avoid the loss of password records due to the capture of the $CH_{i}$, the $CH_{i}$ randomly selects $n_{c}$ nodes among the cluster member nodes and sends the $t_{{arrive_{i} }}^{mob}$ and $Count$ which are collected from the $mob$ to them. If the $mob$ with the same ID reenters the cluster, the $CH_{i}$ and the selected cluster member nodes calculate the difference between the new arrival time and the old arrival time, as well as the new interaction count and the old interaction count value. If the time difference or the interaction count difference exceeds a certain threshold, the $mob$ will be considered a replica node. The cluster head broadcasts the ID of the $mob$ for network-wide isolation. If neither the time difference nor the interaction count difference exceeds a certain threshold, the cluster head node updates the passphrase. The $CH_{i}$ will store the new arrival time of the $mob$ to the cluster and the new number of times the $mob$ interacts with the $CH_{i}$. The new number of times is $Count + 1$. The nodes in the cluster delete the original records, and the $CH_{i}$ re-selects several nodes among the cluster member nodes to store the new arrival time and the new number of interactions. If $t_{{arrive_{i} }}^{mob}$ and $Count$ sent by the $mob$ is validated by the $CH_{i}$ and the selected cluster member nodes, the $CH_{i}$ can calculate the public key $PK_{{ID_{mob} }}^{{}} = f(ID_{mob} )$ of the $mob$ based on the $ID_{mob}$, thus obtaining ${\mathrm{Re}} q,T_{m}$. The $CH_{i}$ verifies whether the data is collected based on ${\mathrm{Re}} q$. If the verification fails, the message is ignored. Otherwise, the $CH_{i}$ will substitute the $ID_{mob}$ stored in the temporary list into its ternary symmetric polynomial to obtain the symmetric key $K_{{{\mathrm{CH}}_{i} }}$ between the two points. The equation is as follows:

5 $$K_{{CH_{i} }} = {\mathrm{f}}_{{1}} (ID_{mob} |C_{mob} ,C_{{CH_{i} }} ,ID_{{CH_{i} }} |C_{{CH_{i} }} )$$

Where $C_{{CH_{i} }}$ denotes the cluster number where the $CH_{{\mathrm{i}}}$ is located; for each replica node detection cycle, the $CH_{{\mathrm{i}}}$ knows its cluster number $C_{{CH_{i} }}$; ${\mathrm{C}}_{{{\mathrm{m}}ob}}$ denotes the cluster number where the $mob$ is currently located. When the $mob$ enters a certain cluster, the cluster number is the same as the cluster number where the $CH_{{\mathrm{i}}}$ is located, i.e., $C_{mob} = {\mathrm{C}}_{{CH_{i} }}$.

Subsequently, the $CH_{{\mathrm{i}}}$ constructs and sends an answer signal with a symmetric key-encrypted message $Mes_{2}$ to the $mob$, as depicted in the following Eq. (6):

6 $${\mathrm{M}}es_{2} = \{ ID_{{CH{}_{i}}} ,C_{{CH_{i} }} ,Count + 1,SIG_{{{\mathrm{SK}}_{{{\mathrm{ID}}_{{CH_{i} }} }} }} (K_{{CH_{i} }} ((DATA_{i}^{{{\mathrm{N}}_{{\mathrm{i}}} }} ,T_{i} )||ID_{{CH_{i} }} ))\}$$

Where $ID_{{CH_{i} }}$ signifies the identity of the $CH_{{\mathrm{i}}}$; $SIG_{{SK_{{ID_{{CH_{i} }} }} }}$ denotes the content that is digitally signed by the private key of the $CH_{{\mathrm{i}}}$; $K_{{CH_{i} }}$ indicates the symmetric encryption process performed by the $CH_{{\mathrm{i}}}$ on the sent message $((DATA_{i}^{{{\mathrm{T}}_{{\mathrm{i}}} }} ,T_{i} )||ID_{{CH_{i} }} )$; $DATA_{i}^{{T_{i} }}$ signifies the information stored in the Bloom filter of the $CH_{i}$ of the $T_{i} - th$ replica node detection cycle; ${\mathrm{T}}_{i}$ denotes the detection cycle number of the node in the cluster. (3) After receiving the message $Mes_{2}$, the mobile node $mob$ initially checks whether it has received ID from the cluster head node $CH_{{\mathrm{i}}}$ according to $ID_{{{\mathrm{CH}}_{i} }}$. If it has received it, it does not process the $Mes_{2}$ with a digital signature; if it has not received it, the $mob$ will records $ID_{{{\mathrm{CH}}_{i} }}$ and $C_{{CH_{i} }}$ in the temporary list. Then, the $mob$ substitutes $ID_{{{\mathrm{CH}}_{i} }}$ and $C_{{CH_{i} }}$ into its ternary symmetric polynomial to derive the symmetric key $K_{mob}$ between the two points, and $K_{mob}$ can be obtained by Eq. (7) as follows:

7 $${\mathrm{K}}_{mob} = {\mathrm{f}}_{{1}} (ID_{{CH_{i} }} |C_{{CH_{i} }} {,}C_{mob} ,{\mathrm{ID}}_{mob} |C_{mob} )$$

The mobile node and the cluster head node belong to the same cluster, i.e., $C_{mob} = C_{{CH_{i} }}$. According to the symmetry property of ternary symmetric polynomials, combining Eqs. (5) and (7), we get $K_{mob} = K_{{CH_{i} }}$. The mobile node $mob$ uses $K_{mob}$ to decrypt the $K_{{CH_{i} }} ((DATA_{i}^{{{\mathrm{T}}_{{\mathrm{i}}} }} ,T_{i} )||ID_{{CH_{i} }} )$. After decrypting, the $mob$ can compute the public key $PK_{{ID_{{CH_{i} }} }}^{{}} = f(ID_{{CH_{i} }} )$ of the $CH_{i}$ according to $ID_{{CH_{i} }}$, thus obtaining $DATA_{i}^{{T_{i} }} ,T_{i}$. Comparing $T_{i}$ with $T_{m}$, if $T_{i} = T_{m}$ is found, it can be inferred that the period number for collecting information by the $mob$ is the same as the period number detected by the replica node. In this case, the $mob$ will store the received $Count + 1,T_{i} ,DATA_{i}^{{T_{i} }}$. If $T_{i} \ne T_{m}$ is found, the information collected by the $mob$ is outdated, and the network has performed the update of the replica node detection cycle. Then, the $mob$ will clear the information in its Bloom filter, store $T_{i}$, and update the sent message $Mes_{1}$. The new equation for $Mes_{1}$ is as follows:

8 $${\mathrm{M}}es_{1} = \{ ID_{mob} ,t_{{arrive_{{\mathrm{i}}} }}^{mob} ,Count + 1,SIG_{{{\mathrm{SK}}_{{{\mathrm{ID}}_{{{\mathrm{mo}}b}} }} }} (({\mathrm{Re}} q,T_{i} )||ID_{mob} )\}$$

After communication between the mobile node and the cluster head node, the mobile node collects the information of different cluster head nodes and can determine replica nodes. If the information collected by the cluster head node reports different geographic locations for the same ID, it indicates that the node associated with that ID is a replica. Then, the mobile node will broadcast and isolate this ID. Meanwhile, the base station can also determine whether the mobile node is a replica node by checking whether the number of interactions between the mobile node and the cluster head node within a certain time exceeds a threshold.

### Mobile inter-node communication phase

**Fig. 4:**
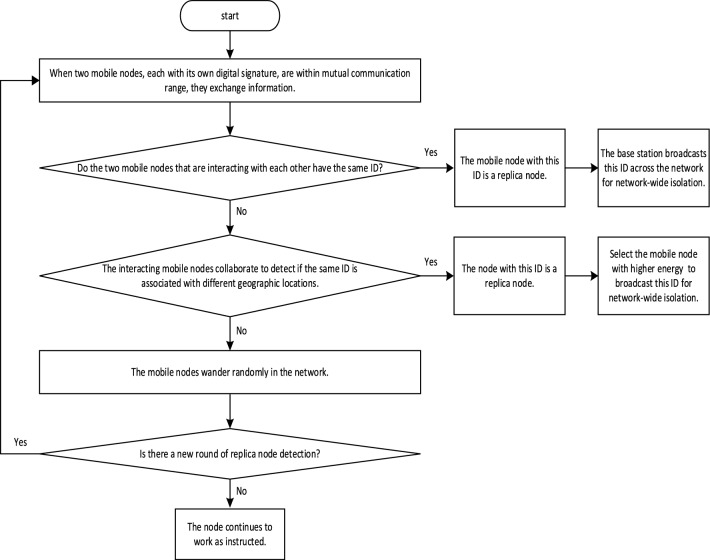
The flowchart of the mobile inter-node communication phase of the KN-MCDP.

Figure 4 is the flowchart of the mobile inter-node communication phase of the KN-MCDP. In the network, the mobile node $mob_{1}$ sends message $\{ ID_{{mob_{1} }} ,t_{{arrive_{{\mathrm{i}}} }}^{{mob_{1} }} ,Count_{{mob_{1} }} ,SIG_{{{\mathrm{SK}}_{{{\mathrm{ID}}_{{{\mathrm{mo}}b_{1} }} }} }} (({\mathrm{Re}} q_{1} ,T_{{m_{1} }} )||ID_{{mob_{1} }} )\}$, and the mobile node $mob_{2}$ sends message $\{ ID_{{mob_{2} }} ,t_{{arrive_{{\mathrm{i}}} }}^{{mob_{2} }} ,Count_{{mob_{2} }} ,SIG_{{{\mathrm{SK}}_{{{\mathrm{ID}}_{{{\mathrm{mo}}b_{2} }} }} }} (({\mathrm{Re}} q_{2} ,T_{{m_{2} }} )||ID_{{mob_{2} }} )\}$, respectively. When the two mobile nodes are in each other’s communication coverage area, they ignore each other’s $t_{{arrive_{i} }}^{mob}$ and $Count$. If $ID_{{mob_{1} }} = ID_{{mob_{2} }}$, it can be determined that the mobile node corresponding to this ID is a replica node, and the base station broadcasts the mobile node with this ID across the network for network-wide isolation. If $ID_{{mob_{1} }} \ne ID_{{mob_{2} }}$, the node can utilize the other party’s ID to obtain the other party’s digitally signed public key. For example, the mobile node $mob_{2}$ can compute the public key $PK_{{ID_{{mob_{1} }} }}^{{}} = f(ID_{{mob_{1} }} )$ of the mobile node $mob_{1}$ based on $ID_{{mob_{1} }} ,$ thus obtaining ${\mathrm{Re}} q_{1} ,T_{{m_{1} }} .$ Similarly, the mobile node $mob_{1}$ can obtain ${\mathrm{Re}} q_{2} ,T_{{m_{2} }}$ based on $ID_{{mob_{2} }} .$ At the beginning, the mobile nodes are time-synchronized with each other, and they store the same replica node detection cycle number, i.e., $T_{{m_{1} }} = T_{{m_{2} }}$. The probability that the network cluster head node resides within the communication radius of the mobile node significantly exceeds the probability that mobile nodes are within each other’s communication radius before their interaction. Consequently, mobile nodes must engage in communication with the network cluster head nodes to exchange and update the detection cycle. Therefore, after a period of time, the replica node cycle number stored by the mobile node $mob_{1}$ remains equal to the replica node cycle number stored by the mobile node $mob_{2}$, i.e., $T_{{m_{1} }} = T_{{m_{2} }}$. In other words, the mobile node between the mobile node should only verify whether ${\mathrm{Re}} q_{1}$ and ${\mathrm{Re}} q_{2}$ pass or not. If the verification fails, the message is ignored. Otherwise, the node sends the information received in the Bloom filter to the other side. When it is found that the intersection of the received message and its message is an empty set, the received message will be saved in the Bloom filter. When it is found that there are different geographic location information corresponding to the same ID in the received information, the mobile node with the higher energy is selected to broadcast and isolate this ID across the network.

## KN-MCDP protocol analysis

### Security analysis of cluster head nodes

In this section, we will discuss the security of the cluster head node under standard and enhanced replica node attacks.

In the KN-MCDP protocol, cluster head nodes collect information from cluster member nodes and send it to the mobile nodes. If the cluster head node is captured, it will cause serious damage to the network. Thus, it is necessary to analyze the security of cluster head nodes.

In this study, the security of cluster head nodes mainly focuses on their ability to resist attacks from replica nodes. In each round of the replica node detection cycle, after the cluster head nodes are formed, a ternary symmetric polynomial is generated. Then, each cluster head node binds its information $ID_{{CH_{i} }} |C_{{CH_{i} }}$ to the generated ternary symmetric polynomial. During each replica node detection cycle, the cluster head node $CH_{{\mathrm{i}}}$ knows its cluster number $C_{{CH_{i} }}$, so $ID_{{CH_{i} }} |C_{{CH_{i} }}$ can be calculated.

After binding, the ternary symmetric polynomial reduces to a binary symmetric polynomial. This binary symmetric polynomial is shown in Eq. (9). For the adversary, ${\mathrm{f}}_{1} (x,y)$ is a constant, x and y are known quantities, and ${\mathrm{b}}_{{{\mathrm{ij}}}}$ is an unknown variable that requires a solution.

9 $${\mathrm{f}}_{1} (x,y) = \sum\limits_{i,j = 0}^{t} {b_{ij} } x^{i} y^{j}$$

Under standard attack conditions, for $$f_{1} (x,y)$$, it is a $$(t + 1)^{2}$$ dimensional linear equation. Since it is a symmetric polynomial, i.e., $$b_{ij} = b_{ji} (0 \le i \le {\mathrm{t}},0 \le {\mathrm{j}} \le t),$$$$f_{1} (x,y)$$ can be reduced to a $$(t + 1)(t + 2)/2 -$$ dimensional linear equation. To solve the $$(t + 1)(t + 2)/2$$ dimensional linear equation, it is necessary to have $$(t + 1)(t + 2)/2$$ linear equations with the same variable forming a system of equations, and the coefficient determinant cannot be 0. Therefore, to derive the primitive ternary $$t$$ degree polynomial, the adversary must capture at least $$(t + 1)*$$$$(t + 2)/2$$ nodes, implying that the cluster head node can resist node capture attacks is $$(t + 1)(t + 2)/2$$. In practice, the KN-MCDP protocol can enhance the ability of cluster head nodes to resist replica node attacks by controlling $$t$$, ensuring the security of cluster head nodes.

Under enhanced replica node attacks, adversaries may launch side-channel attacks or attempt to leak partial node information. To counter side-channel threats, our approach can leverage existing lightweight anti-side-channel protection techniques. For instance, when implementing polynomial evaluation, the KN-MCDP protocol can employ constant-time algorithms^44^ to eliminate timing information leaks or use random masking techniques^45^ to obscure the correlation between intermediate values and power consumption. As for the partial leakage of a node’s internal information, the algebraic properties of polynomials guarantee that recovering a t-degree polynomial requires a set of $(t + 1)(t + 2)/2$ coefficients. Therefore, partial leakage of node information does not compromise the secret. Acquiring only a subset of coefficients is analogous to solving an underdetermined system of linear equations, which cannot yield a unique solution for the original polynomial. Therefore, such partial leakage does not compromise the threshold security of the system.

### Communication overhead and storage overhead

There are two metrics for measuring the efficiency of a replica node detection protocol: the communication overhead and the storage overhead. The two metrics are defined as follows:

1. Communication overhead: the total number of packets sent and received by the node in one epoch.
2. Storage overhead: the average number of validation messages that each node should store to complete the detection.

#### Communication overhead

The communication overhead is divided into two parts. One part is the communication overhead incurred for the cluster head nodes and intra-cluster nodes. The other part is the communication overhead incurred for the mobile nodes.

The communication overhead incurred for the intra-cluster node and cluster head node includes the costs of a message sent from an intra-cluster node to the relevant cluster head, and from the cluster head to the mobile node, respectively. Since the total number of static nodes in the network is $N_{s}$, the communication overhead incurred for the intra-cluster node and cluster head node is $O(N_{s} )$.

**Fig. 5:**
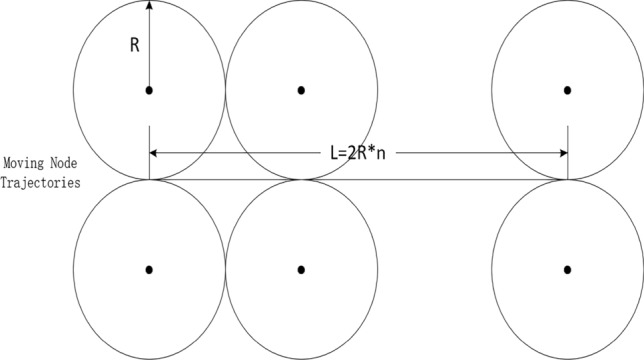
Schematic of the maximum number of clusters.

**Fig. 6:**
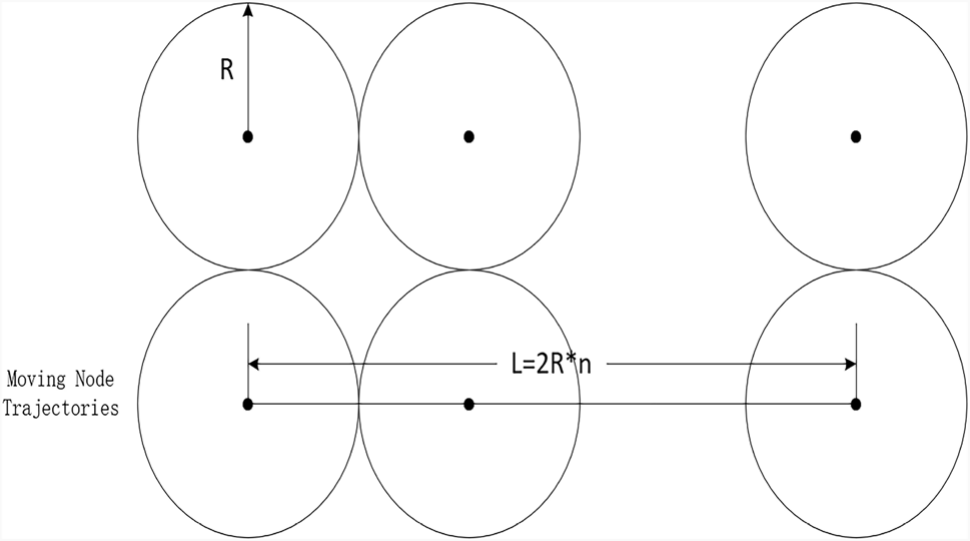
Schematic of the minimum number of clusters.

The communication overhead incurred for the mobile node includes the overhead incurred for the mobile node to collect information about the cluster head nodes and the communication overhead incurred for the mobile node to roam randomly. Since the communication overhead incurred for a mobile node to reach each cluster is the same (according to the description of the KN-MCDP scheme, it is $2n_{c} + 4$), the communication overhead incurred for a node in one epoch depends on the number of clusters it traverses. At this point, there may be two extreme cases: (1) the mobile node’s trajectories during one epoch all pass through the edges of the clusters it reaches, as illustrated in Fig. 5; (2) the mobile node’s trajectories in one epoch all pass through the center of the reached cluster, as depicted in Fig. 6. The average distance moved by a node in one epoch is $L$. The maximum value of the number of clusters it passes through is $L/R + 2$, and the minimum value is $L/2R + 1$. Therefore, the communication overhead incurred for each mobile node in one epoch is between $(2n_{c} + 4)(L/R + 2)$ and $(2n_{c} + 4)(L/2R + 1)$. Since the number of mobile nodes in the network is $N_{m}$, it can be inferred that the traffic generated by the mobile node collecting information in one epoch should be between $N_{m} (2n_{c} + 4)(L/R + 2)$ and $N_{m} (2n_{c} + 4)(L/2R + 1)$. Here, $L/R$ is not greater than some constant and $n_{c}$ is a relatively limited constant, so the communication overhead incurred for the mobile nodes is $O(N_{m} )$.

The communication overhead incurred for the intra-cluster node and cluster head node is $O(N_{s} )$, while that incurred by mobile nodes is $O(N_{m} )$. By considering the overhead associated with cluster head nodes, intra-cluster nodes, and mobile nodes collectively, the total communication overhead of the network is determined to be $O(N)$.

#### Storage overhead

Since the KN-MCDP scheme is a collaborative storage scheme, its storage overhead is divided into the storage overhead of static nodes and the storage overhead of mobile nodes.

The storage overhead of static nodes is analyzed first. The static nodes are subdivided into cluster head nodes and cluster member nodes. The cluster head node needs to store an $N_{m} \times d$ ($d$ is the neighborhood degree of the static node); this part of the cluster head node occupies a storage space unit of $N_{m} d$. Furthermore, the cluster head node stores a two-variable t-degree polynomial ${\mathrm{f}}_{1} (x,y)$, which occupies a storage space in a unit of $(t + 1)^{2}$. Since $b_{ij}$ has a symmetry, the actual storage unit occupied by this component is $(t + 1)(t + 2)/2$. Therefore, the total storage overhead occupied by the cluster head node is $N_{m} d + (t + 1)(t + 2)/2$.

Subsequently, the storage overhead of cluster member nodes is analyzed. The probability that the cluster member nodes are selected as a storage object when one mobile node arrives at the cluster is $n_{c} /d$. The selection events are independent of each other. Therefore, when $N_{m}$ mobile nodes arrive at the cluster sequentially, the storage object selection event can be considered as $N_{m}$ independent Bernoulli trials, which follow a binomial distribution with parameters $N_{m} ,n_{c} /d$. Consequently, the average value of the storage overhead of the cluster member nodes is $N_{m} \cdot n_{c} /d$.

The storage overhead of mobile nodes is analyzed. Since the number of nodes in the static network is $N_{s}$ and the neighborhood degree of the node is $d$, the number of clusters in the network can be approximated as $N_{s} /(d + 1)$. To complete the detection, each mobile node must store as $1 \times N_{s} /(d + 1)$. Moreover, the mobile node should also store a two-variable t-degree polynomial ${\mathrm{f}}_{1} (x,y)$, which occupies a storage unit of $(t + 1)(t + 2)/2$. Therefore, the total storage overhead occupied by the mobile node is $(t + 1)(t + 2)/2 + N_{s} /(d + 1)$.

In summary, since $d$ and $t$ are not greater than some constant and $n_{c}$ is a relatively limited constant, the storage overhead of static nodes in the KN-MCDP scheme is $O(N_{m} )$, and the storage overhead of mobile nodes is $O(N_{s} )$. The network’s storage overhead includes both the storage required for static nodes and the storage required for mobile nodes. Thus, the storage overhead of the network is $O({\mathrm{N}})$.

As mentioned earlier, there are a total of $N$ nodes in the network. During a round of replica node detection, $n$ cluster head nodes are formed in the network. To analyze the performance of the KN-MCDP protocol, this study compares it with the BS protocol and the RM protocol proposed by Parno et al. in 2007, the CBDM protocol proposed by H. Zhou et al. in 2014, and the BFCP protocol proposed by J. Cheng in 2023. The results are compared in Table 1.

**Table 1:** Communication overhead and storage overhead comparison.

Agreement name	Communications overhead	Storage overhead
BS	$O(N^{2} )$	$O(N)$
RM	$O(N^{2} )$	$O(\sqrt N )$
CBDM	$O(N)$	$O(n)$
BFCP	$O(N)$	$O(n)$
KN-MCDP	$O(N)$	$O(N)$

As depicted in Table 1, (1) CBDM, BFCP, and KN-MCDP perform local detection utilizing cluster head nodes before global detection, so their communication overhead is much lower than that of BS and RM. (2) The CBDM protocol directly accesses the information of nodes in the cluster, whereas the BFCP protocol and the KN-MCDP protocol store node information by using Bloom filters. For both the BFCP and KN-MCDP protocols, when k = 1, the storage method is the CBDM protocol. In practice, k must be a positive integer greater than 1, so we can control k to achieve higher storage utilization and reduce the storage overhead of the network. (3) For the BFCP protocol, as long as no replica node is detected, the cluster head node will communicate with neighboring cluster heads continuously. Eventually, each cluster head is likely to store information about all nodes in the network using Bloom filters, thereby increasing the storage burden on the cluster head node. However, in the KN-MCDP protocol, the cluster head node that carries the Bloom filters is responsible only for collecting information about nodes within the cluster. This reduces the storage overhead of the cluster head node.

**Table 2:** Simulation parameters table.

**Simulation parameters**	**Configuration**
Simulator	NS-2
Simulation network area	100 m × 100 m
Number of static nodes	50,60,70,80,90,100
Total number of replica nodes	2,3,4,5,6
Total number of mobile nodes	1,2,3,4
Static node communication radius	10 m
Mobile node communication radius	15 m
Initial energy of static nodes	100 J
Initial energy of mobile nodes	1000 J
The model adopted for mobile node motion	Random Direction model
Types of network attacks	Replica node attack
Velocity of mobile nodes	[2, 20]m/s
Dwell time of mobile nodes	[0,5] s
Replica node detection cycle	30 s
Bloom filter size at the cluster head node	60 b
Bloom filter size at the mobile node	500,600,700,800 b
Number of hash functions	3,4,5,6,7
Degree of a ternary symmetric polynomial	2,3,4,5
Number of replicates per experiment	20

### Energy consumption balance analysis

Compared to the BS, CBDM, and BFCP protocols, the additional cryptographic overhead introduced by KN-MCDP primarily stems from the digital signature, the ternary symmetric polynomial evaluation, and the symmetric encryption. Among these, the digital signature incurs the highest computational overhead each time a mobile node establishes a new session with a cluster head node. However, once a session is established, repeated signatures are not required for the duration of that session. The evaluation of ternary symmetric polynomials is performed twice during each session establishment (once by each communicating party) to derive the shared key. Symmetric encryption is an inherently low-energy operation. Therefore, overall, the overhead of KN-MCDP cryptography is relatively low.

KN-MCDP uses Bloom filters to store and compress data, significantly reducing network storage overhead and energy consumption for communication. By leveraging cluster head nodes, several high-energy mobile nodes, and the base station to identify and isolate replica nodes, it balances network load, prevents premature node failure from excessive forwarding, and extends network lifespan. Furthermore, key parameters of the protocol, such as the polynomial degree, Bloom filter size, and the number of hash functions, are adjustable. During actual deployment, we can optimize these parameters based on the network’s security requirements, node resources, and expected lifetime.

Therefore, from a quantitative perspective, we found that KN-MCDP achieves fundamental optimization of the communication architecture by strategically introducing lightweight cryptographic operations, thereby reducing overall energy consumption and extending network lifetime.

## Experimental results

To provide a more intuitive analysis of the KN-MCDP protocol’s performance, this paper designs comparative experiments to benchmark it against three other protocols: BS, CBDM, and BFCP. The four protocols employ distinct detection principles. BS is a non-clustered centralized detection scheme; CBDM relies on cluster heads; BFCP utilizes Bloom filters; and KN-MCDP integrates static clustering with dynamic coordination. Despite these architectural differences, their core objectives remain consistent. All are dedicated to addressing replica node attacks in wireless sensor networks and uniformly select detection rate, computational overhead, and network lifetime as primary evaluation metrics. During the simulation phase, all protocols are deployed in an identical network environment, encompassing consistent physical node deployment schemes and attack model configurations. This ensures the fairness and rigor of the experiments. Any observed performance differences can be directly attributed to the inherent design architectures and algorithmic logic of each protocol.

### Simulation parameter settings

The proposed KN-MCDP protocol is evaluated through simulations on the NS-2 platform under the following setup. $N_{s}$ sensor static nodes and $N{}_{{\mathrm{m}}}$ mobile nodes are randomly deployed within a 100 × 100 m^2^ square area. The communication radius of the static nodes is 10 m, the initial energy is 100 J, and the detection period of one round of replica nodes is 30 s. The cluster head node carries the Bloom filter address sequence of length m = 60, and each element corresponds to k = 5 mutually independent hash functions. The degree of the ternary symmetric polynomial generated by the cluster head node is $t$. The communication radius of the mobile nodes is 15 m, and the initial energy is 1000 J. They carry a Bloom filter of length M, which also uses k = 5 mutually independent hash functions. Each mobile node generates a ternary symmetric polynomial of degree $t$. The mobile nodes move in the detection area according to the Random Direction model with a speed of $v = 2\sim 20m/s$ and a dwell time of $0\sim 5s$. To minimize errors, the experiments are repeated 20 times, and the results are averaged. All key simulation parameters are summarized in Table 2.

### Detection rate

#### The effect of mobile node speed on detection rate

**Fig. 7:**
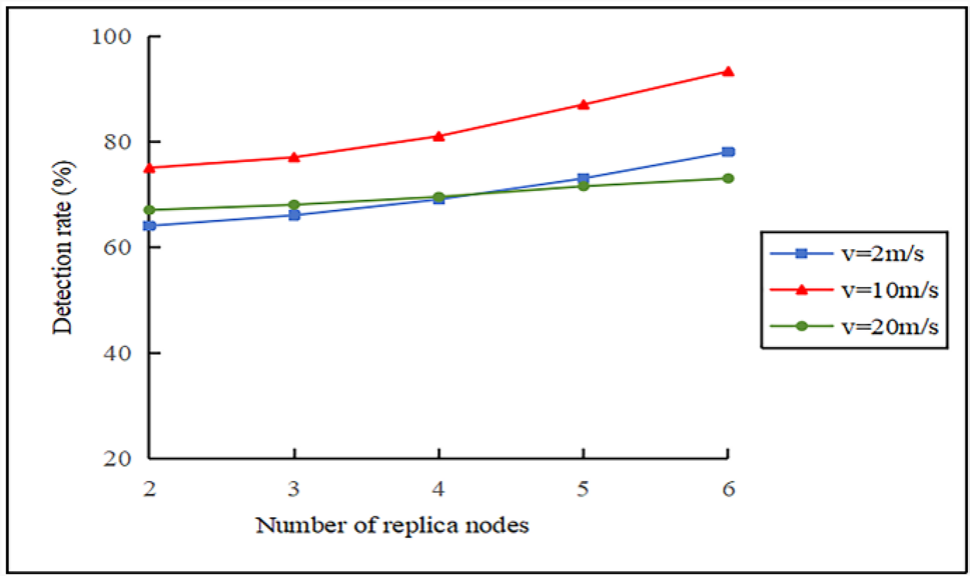
Effect of the number of mobile nodes on the detection rate.

Figure 7 depicts the effect of the mobile node speed on the detection rate. When $v = 2 \, m/s$, the communication link between the mobile node and the cluster head node remains stable, the detection rate increases steadily as the number of replica nodes increases. However, due to the slow speed, the mobile node’s network coverage is an issue. For $v = 20 \, m/s$, the detection rate is initially higher than that of $v = 2 \, m/s$. This is because faster movement allows for quicker encounters with cluster head nodes. However, when the number of replica nodes exceeds 4, the detection rate is lower than that of $v = 2 \, m/s$. This occurs because connection issues impose a stringent constraint at elevated speeds, and increasing the number of replica nodes has a limited effect on improving the detection rate. When $v = 10 \, m/s$, the coverage and connectivity issues are relatively balanced, achieving a high detection rate. Therefore, in the subsequent experiments, the average speed of the Random Direction model can be set to $\mathop v\limits^{\_} = 10 \, m/s$.

#### Impact of the number of mobile nodes and replica nodes on the detection rate

**Fig. 8:**
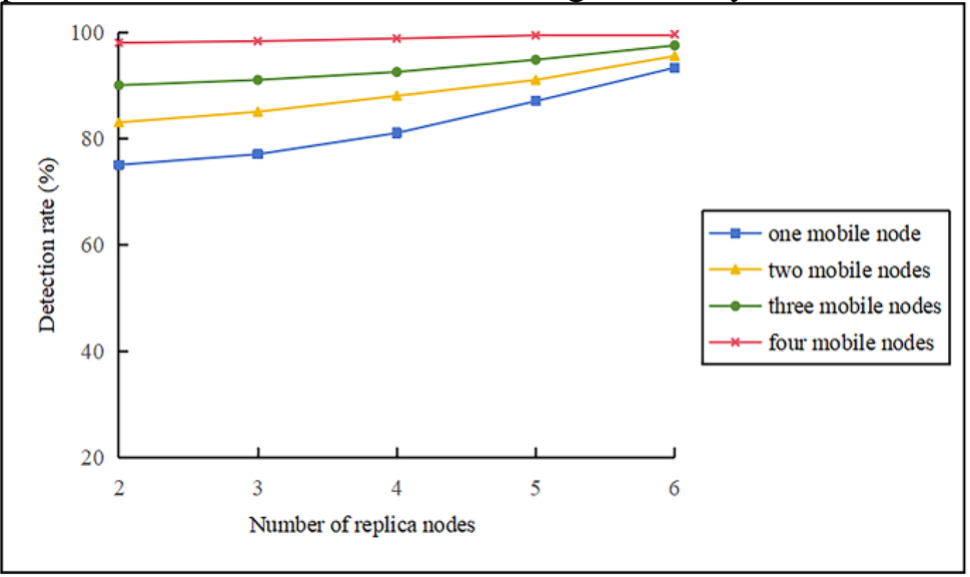
Effect of the number of mobile nodes and replica nodes on the detection rate.

Figure 8 depicts the effect of the number of mobile nodes and replica nodes on the detection rate when the average speed of the mobile nodes is $\mathop v\limits^{\_} = 10 \, m/s$. The following observations can be made: (1) When the same replica nodes exist in the network, the detection rate increases significantly with each additional mobile node. As the number of mobile nodes increases, the probability of a mobile node meeting either a cluster head or another mobile node increases significantly. Even if one mobile node produces a false positive due to the Bloom filter, the other mobile nodes will compensate for it. (2) The KN-MCDP protocol is highly sensitive to an increasing number of replica nodes. When the number of mobile nodes remains constant, adding more replica nodes causes a significant increase in detection probability. This is because, as the number of replica nodes increases, the mobile node is more likely to gather information from them. Even if one node fails to detect an item due to a false positive, the other replica nodes will compensate for it. Therefore, for the KN-MCDP protocol, adding a limited number of mobile nodes and replica nodes to the network will significantly increase its detection rate.

#### The effect of Bloom filter size on detection rate

**Fig. 9:**
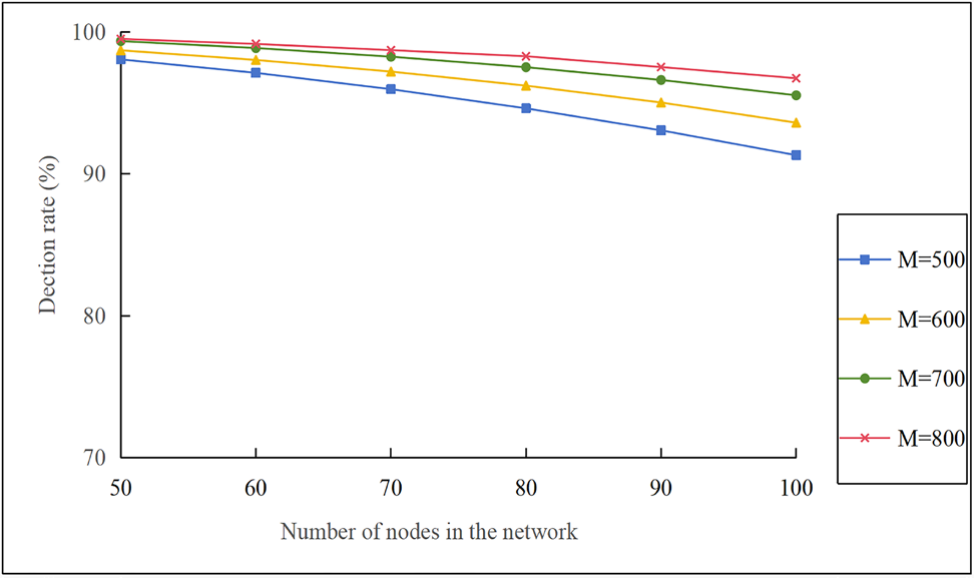
The effect of Bloom filter size on detection rate.

Figure 9 presents the effect of Bloom filter size at the mobile node on the detection rate for $\mathop v\limits^{\_} = 10 \, m/s$ and $N_{m} = 3$. The following observations can be made: (1) When mobile nodes carry the same number of bits M in their Bloom filters, the detection rate of replica nodes decreases as the number of network nodes increases. As shown by Eq. (3) in this study, when M and k are constant, a larger number of network nodes leads to a higher false positive rate $p$ in the Bloom filter, thereby reducing the detection rate. (2) When the number of network nodes is the same, the higher the number of bits M carried by the mobile node in the Bloom filter, the higher the detection rate of replica nodes. This is because, as shown in Eq. (3), the larger M is, the lower the false positives in the Bloom filter, and the higher the detection rate. Additionally, the network employs three mobile nodes to collaboratively detect replica nodes. Even if one node fails to detect an item due to a false positive, the other nodes will compensate for it. This mutual collaboration further enhances the detection rate of replica nodes. (3) The larger M is, the greater the storage overhead for the cluster head node. As shown in Fig. 9, the detection rate is already quite high at M=700. Therefore, for the subsequent discussion of other metrics in this paper, we can set M=700 as the baseline.

#### Effect of the number of nodes on the detection rate

**Fig. 10:**
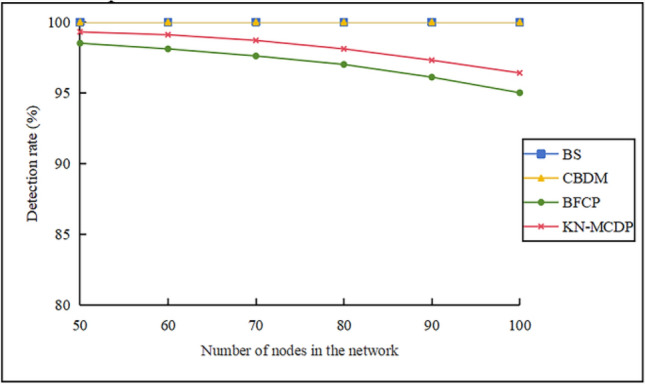
Effect of the number of nodes on the detection rate.

Figure 10 presents the effect of the number of network nodes on the detection rate for $\mathop v\limits^{\_} = 10 \, m/s$ , $N_{m} = 3$ and ${\mathrm{M}} = 700$. The BS and CBDM exhibit superior performance, whereas the KN-MCDP ranks second- best, and BFCP ranks last. This pattern can be explained as follows: (1) As the number of static nodes increases from 50 to 100, the detection rates of the BS protocol and CBDM protocol remain at 100%. The KN-MCDP protocol decreases from approximately 99% to around 97%, while the BFCP protocol drops from about 98% to roughly 95%. (2) BS and CBDM collect information from all nodes in the network through the resourceful base station, resulting in a high detection rate. However, as analyzed earlier in this study, the BS protocol suffers from single-point-of-failure issues at the base station and network load imbalance, resulting in poor performance metrics such as scalability, energy consumption, and network lifetime. For CBDM, it adopts a local-to-global approach where all cluster head nodes transmit information to the base station. This local-to-global approach also inevitably introduces high communication overhead between the base station and nearby nodes in centralized schemes, thereby impacting the network lifetime. (3) The cluster head nodes and the mobile nodes that collect information in the KN-MCDP protocol show a reduced detection rate due to the presence of false positives in the Bloom filters. However, the overall detection rate of the KN-MCDP protocol is still high. (4) The detection rate of the KN-MCDP exceeds that of BFCP. This is because the latter uses only cluster heads with limited storage capacity to fuse intra-cluster and network-wide information, making it easy to produce false detections due to the false positives of the Bloom filter. Conversely, KN-MCDP selects several mobile nodes with high energy to collect information from the cluster head nodes and other mobile nodes. Even if one of them generates a false positive due to the Bloom filter, the other mobile nodes will compensate for it.

### Maximum amount of information stored in a single node

**Fig. 11:**
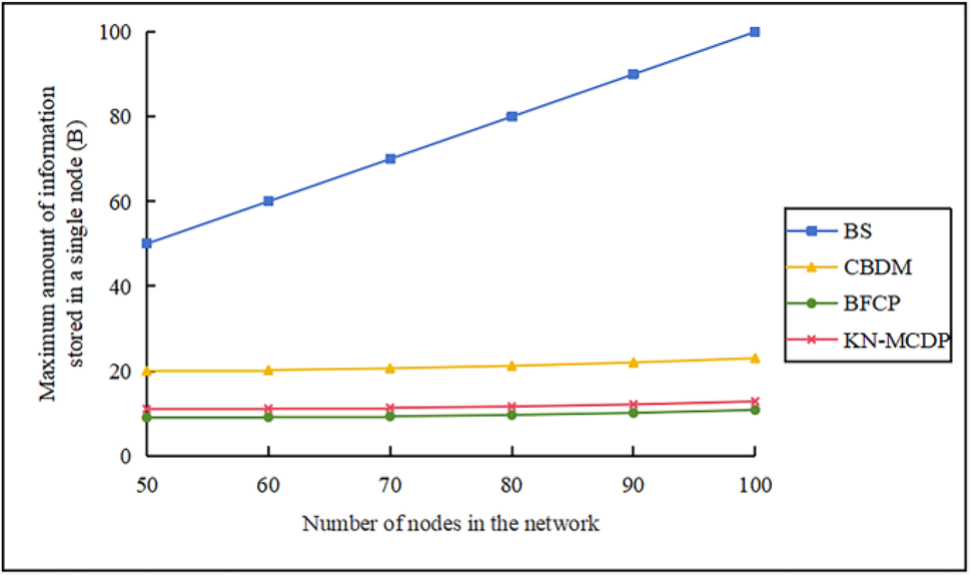
Comparison of the maximum amount of information stored in a single node.

When $\mathop v\limits^{\_} = 10 \, m/s$,$N_{m} = 3$, and ${\mathrm{M}} = 700$, the number of static network nodes is $N_{s} = 100$, and the degree of the symmetric ternary polynomial is $t = 3$, the comparison chart of the maximum amount of information stored by a single node in the network is shown in Fig. 11. The following can be observed:

(1) When the number of static nodes increases from 50 to 100, the maximum storage capacity per node in the BS protocol rises from approximately 50 B to 100 B. The CBDM protocol remains stable at around 20 B, while KN-MCDP and BFCP generally maintain levels of approximately 12 B and 10 B, respectively. (2) The maximum amount of information stored by BS is directly proportional to the number of network nodes. This is because all nodes in the BS protocol have to send relevant information to the base station, resulting in excessive communication and storage burdens on the base station and its nearby nodes. (3) The CBDM, BFCP, and KN-MCDP protocols are less affected by the number of nodes in the network in terms of the maximum amount of information stored in a single node. The reason is that the percentage of cluster head nodes in the network remains unchanged regardless of the total number of nodes. As the number of network nodes increases, the number of cluster heads increases as well. (4) The BFCP and KN-MCDP protocols store less maximum information in a single node than the CBDM protocol. The reason is that the CBDM protocol uses regular storage, whereas the BFCP and KN-MCDP protocols use Bloom filters to store relevant information, thereby enhancing storage efficiency and reducing storage overhead. (5) The KN-MCDP protocol stores slightly more maximum information in a single node than the BFCP protocol because the mobile nodes and cluster head nodes in the KN-MCDP protocol store not only the node information in the network but also the ternary symmetric polynomials.

### Communication overhead

**Fig. 12:**
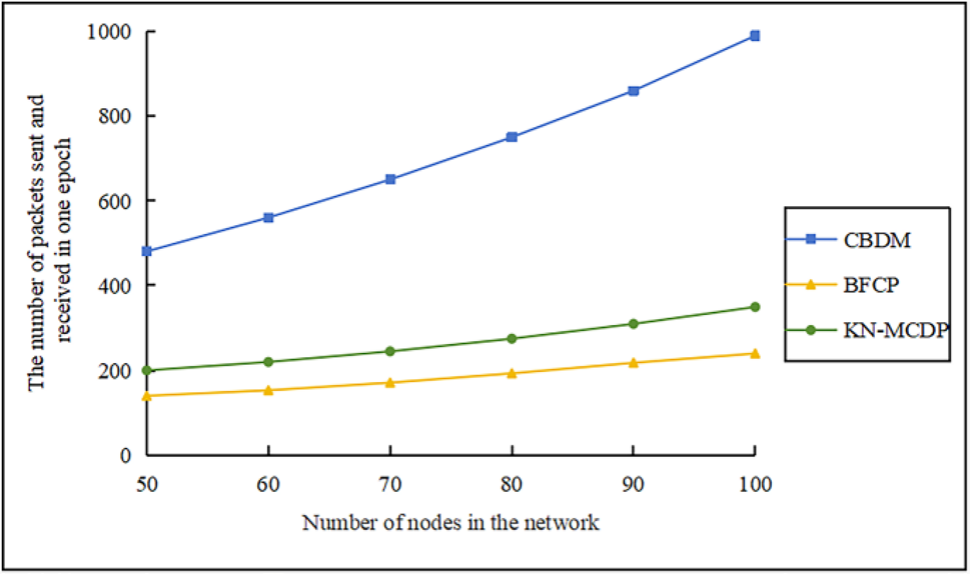
Communication overhead comparison chart.

When $\mathop v\limits^{\_} = 10 \, m/s$, $N_{m} = 3$, ${\mathrm{M}} = 700$,$N_{s} = 100$, and $t = 3$, the communication overhead comparison chart for different protocols is shown in Fig. 12. From the figure, we can observe: (1) As the number of static nodes increases from 50 to 100, communication overhead exhibits a three-tiered differentiation: the BS protocol incurs extremely high overhead, so excessive that it cannot be accommodated within the graph; the CBDM protocol exhibits moderate overhead, increasing from approximately 500 to around 1000; the BFCP and KN-MCDP protocols maintain relatively low overhead levels. Specifically, BFCP increases from approximately 150 to about 200, while KN-MCDP rises from roughly 200 to approximately 300. (2) For the BS protocol, since each node broadcasts messages, the communication overhead of the protocol increases exponentially with the number of nodes, resulting in excessive communication overhead. Therefore, the BS protocol will not be described here. (3) The CBDM protocol adopts a clustered structure, where the cluster head communicates with the nodes within the cluster conventionally, resulting in significant communication overhead for the cluster head nodes. (4) The BFCP protocol and the KN-MCDP protocol use Bloom filters to compress information. This significantly reduces the size of transmitted data. The energy used to transmit information between nodes is much less than what is needed to send complete data lists, leading to low communication overhead. (5) Although both the KN-MCDP protocol and the BFCP protocol utilize Bloom filters to compress transmitted information, the KN-MCDP protocol incurs slightly higher communication overhead than BFCP due to its low-frequency digital signatures and low-energy key exchange.

### Average energy overhead

**Fig. 13:**
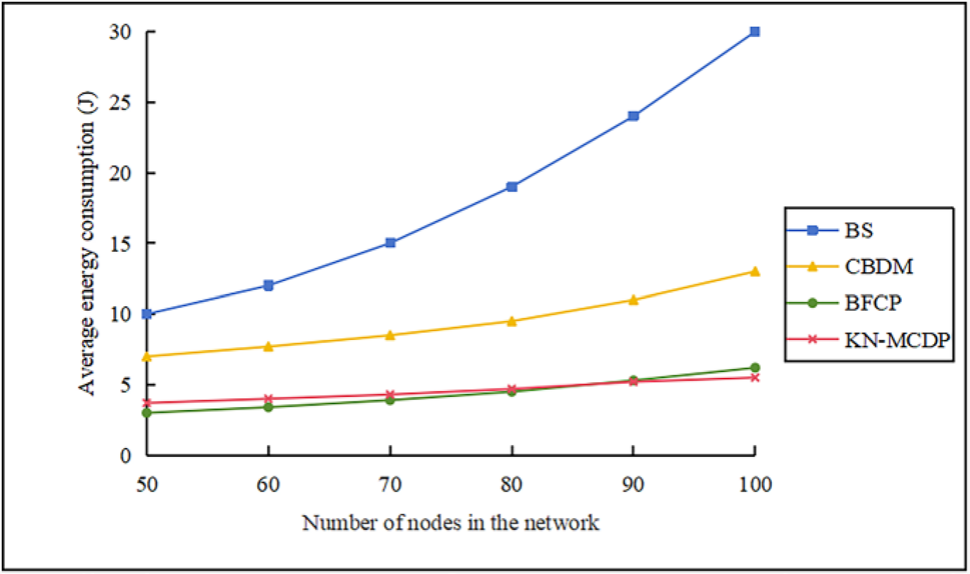
Average energy overhead comparison chart.

When $\mathop v\limits^{\_} = 10 \, m/s$, $N_{m} = 3$, ${\mathrm{M}} = 700$, $N_{s} = 100$, and $t = 3$, the average energy consumption of the nodes is compared as depicted in Fig. 13. The following observations can be made: (1) As the network scale expands, the average energy overhead of each protocol varies differently. The BS protocol exhibits the highest energy consumption, increasing from approximately 10 J to about 30 J. The CBDM protocol rises from approximately 7 J to about 10 J. The BFCP protocol grows from approximately 3 J to about 5 J. The KN-MCDP protocol increases from approximately 3.5 J to about 4 J. (2) In the BS protocol, each node needs to send the corresponding information to the base station, which consumes the most energy, and the curve shows an exponential growth trend. (3) For the CBDM protocol, although the average energy consumption gradually increases as the number of nodes increases, it remains lower than that of the BS protocol due to its clustered architecture. (4) The BFCP and KN-MCDP protocols utilize Bloom filters to store and compress data, significantly reducing network storage and communication overhead. Consequently, their average energy consumption is substantially lower than that of the BS and CBDM protocols. (5) The KN-MCDP protocol generally consumes more energy than the BFCP protocol. This is because the KN-MCDP protocol employs low-frequency digital signatures, ternary symmetric polynomials, and low-energy symmetric key encryption, which slightly increases energy consumption. However, when many network nodes exist, the BFCP protocol repeatedly fuses information between cluster head nodes without detecting replica nodes, resulting in higher energy consumption. Thus, the average energy consumption of nodes in the BFCP protocol may surpass that of the KN-MCDP protocol.

### Network lifetime

#### The impact of the t-value on the network lifetime

**Fig. 14:**
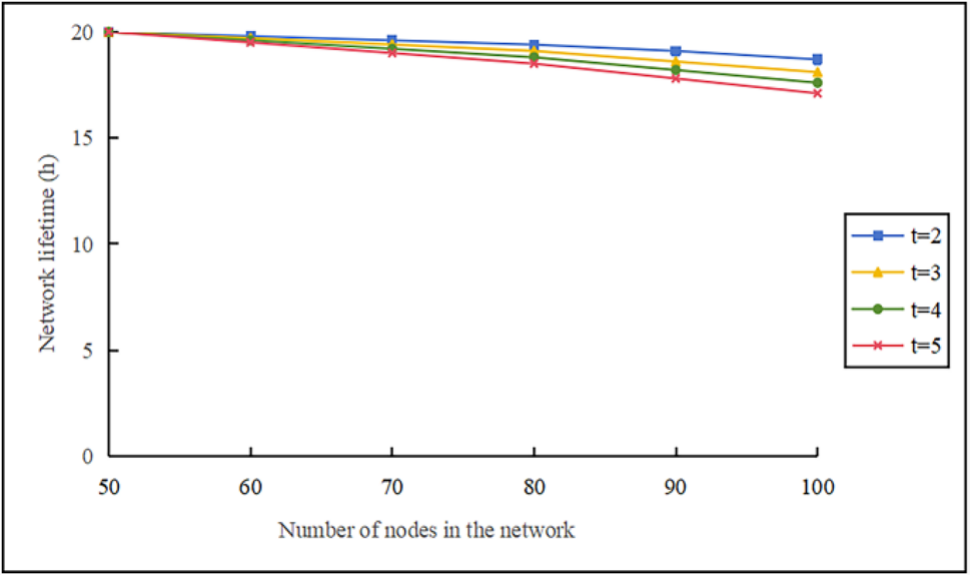
Impact of the t-value on the network lifetime.

Figure 14 illustrates the impact of changes in $t$ values on the network lifetime of the KN-MCDP protocol for $\mathop v\limits^{\_} = 10 \, m/s$, $N_{m} = 3$, and ${\mathrm{M}} = 700$. When the number of nodes in the network is the same, a larger $t$ leads to a shorter network lifetime. This is because the larger the value of $t$, the higher the storage and computational costs, which in turn affect the network lifetime. However, from the previous context, we know that a larger t-value indicates a stronger ability to resist replica nodes. In practical applications, we need to choose $t$ based on specific circumstances to ensure both the security of the cluster head node and the lifetime of the network.

#### Network lifetime comparison chart

**Fig. 15:**
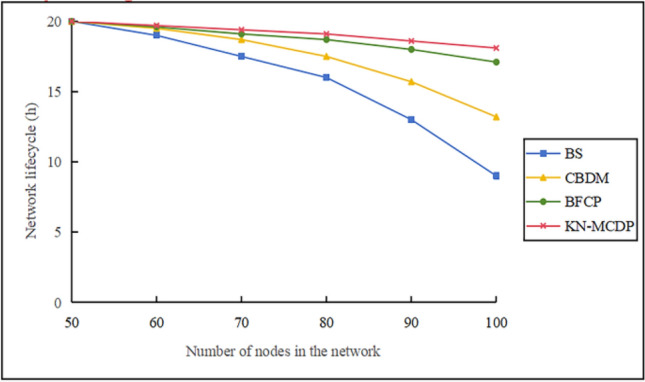
Network lifetime comparison chart.

When $\mathop v\limits^{\_} = 10 \, m/s$, $N_{m} = 3$, $N_{s} = 100$, ${\mathrm{M}} = 700$, and $t = 3$, the network lifetime comparison chart is shown in Fig. 15. BS has the shortest network lifetime, CBDM ranks second, and KN-MCDP has the longest lifetime. This can be explained as follows: (1) Overall, under identical network scales, the BS protocol exhibits the shortest lifespan; the CBDM protocol demonstrates moderate longevity; the BFCP protocol shows extended durability; while the KN-MCDP protocol achieves the longest network lifespan with optimized parameters at t=3. Specifically, as the number of static nodes increases from 50 to 100, the network lifetime of BS decreases from approximately 20 hours to 9 hours, CBDM decreases from approximately 20 hours to 14 hours, BFCP increases from approximately 20 hours to 18 hours, and KN-MCDP with t=3 increases from approximately 20 hours to 19 hours. (2) All nodes in the BS protocol must send the relevant information to the base station, which imposes heavy communication and storage burdens on the base station and the nodes near it, making them exceedingly large. Thus, the BS protocol has the shortest network lifetime. (3) For the CBDM protocol, since the base station collects information from the cluster head nodes rather than from all nodes in the entire network, its network lifetime is longer than that of the BS protocol. (4) For the BFCP protocol, the cluster head node not only collects the information from the nodes within the cluster but also constantly collects information from the nodes across the whole network. The cluster head node of the BFCP protocol has significant communication and storage overhead. Consequently, its network lifetime is shorter than that of the protocol. (5) The KN-MCDP protocol employs infrequent digital signatures, ternary symmetric polynomials, and low-energy symmetric encryption methods. While it moderately increases network computational and energy overhead, it significantly reduces storage and communication costs by utilizing Bloom filters for data storage and compression. By leveraging cluster head nodes, several high-energy mobile nodes, and the base station to identify and isolate replica nodes, it balances network consumption. The KN-MCDP protocol achieves the longest network lifetime because the limited overhead introduced by cryptographic operations is offset by substantial savings in communication overhead, yielding a net benefit.

## Conclusions

This study introduces a multi-point collaborative mobile replica node detection protocol based on key negotiation, referred to as KN-MCDP. The protocol comprises the intra-cluster localized detection phase, the communication phase between mobile nodes and intra-cluster nodes, and the mobile inter-node communication phase. It can not only identify replica nodes in static WSNs but also determine whether a mobile node in the network is a replica node. The protocol prevents information leakage from the cluster head node by encrypting the exchanged information between the mobile node and the cluster head node. It also reduces network storage and communication overhead by using Bloom filters to store information. In different phases, the protocol uses cluster head nodes, mobile nodes, and the base station to identify and isolate replica nodes, thereby balancing the network’s energy overhead and extending its lifetime. The simulation experiments show that the KN-MCDP protocol can achieve a high detection rate with only a few additional mobile nodes. Compared to the BS, CBDM, and BFCP protocols, the KN-MCDP protocol has relatively small storage, communication, and energy overhead, and the longest network lifetime.

However, the KN-MCDP protocol proposed in this study also has certain limitations. First, its design is primarily tailored for hybrid network scenarios in which a limited number of mobile nodes are introduced into an otherwise static wireless sensor network. In practice, however, sensor network deployments may consist entirely of stationary nodes or fully mobile sensor ensembles, contexts in which the KN-MCDP protocol may not be optimally suited. Second, the simulation experiments evaluating the KN-MCDP protocol rely on an idealized link-layer model. This model does not fully account for dynamic real-world wireless channel impairments, such as signal attenuation and multipath fading, which can significantly affect protocol performance and the interpretation of experimental results.

## Data Availability

All data generated or analyzed during this study are included in this published article.

## Abbreviations

*N*: Number of nodes in the network
${N}_{m}$: Total number of mobile nodes in the network
${N}_{s}$: Total number of static nodes in the network
$${C}_{i}$$: Cluster i
*d*: Neighborhood degree of the static node
$CH_{i}$: Cluster head node i
${n}_{c}$: The cluster head randomly selects the number of cluster member nodes and relays the mobile nodes’ information to them
*ID*: Identifier document
*mob*: Mobile node
*K*: Symmetric key
*PK*: Public key
*SK*: Private key
${f}_{1}$(): Ternary symmetric polynomial
*f*(): Public key calculation function
*T*: Replica node detection cycle
${DATA}_{i}^{T}$: Information stored in the Bloom filter of the ${CH}_{i}$ of the T-th replica node detection cycle
$SIG_{{SK_{i} }} (M)$: The content *M* is signed by the private key of node i
${Mes}_{1}$: Message sent by the *mob* to the ${CH}_{i}$
${Mes}_{2}$: Message that the ${CH}_{i}$ replies to the *mob*

## References

[1] Dhanaraj, R. K., Maragatharajan, M., Sureshkumar, A. & Balakannan, S. P. On-device AI for climate-resilient farming with intelligent crop yield prediction using lightweight models on smart agricultural devices. *Sci. Rep.* **15**, 31195. 10.1038/s41598-025-16014-4 (2025). 40854942 10.1038/s41598-025-16014-4 PMC12378187

[2] Alshahrani, A. et al. Internet of things driven object detection framework for consumer product monitoring using deep transfer learning and hippopotamus optimization. *Sci. Rep.* **15**, 30109. 10.1038/s41598-025-13224-8 (2025). 40820124 10.1038/s41598-025-13224-8 PMC12358576

[3] Wang, X. et al. PIA-A secure and efficient identity authentication scheme in telemedicine via the PUF method. *Sci. Rep.* **15**, 6846. 10.1038/s41598-025-89502-2 (2025). 40011568 10.1038/s41598-025-89502-2 PMC11865531

[4] Gouda, B. S. et al. Distributed fault detection in sparse wireless sensor networks utilizing simultaneous likelihood ratio statistics. *Pervasive Mob. Comput.* **110**, 102043. 10.1016/j.pmcj.2025.102043 (2025).

[5] Frahtia, A., Benssalah, M. & Drouiche, K. An efficient UHF RFID tag estimation technique based on belief function theory. *Int. J. Commun. Syst.* **38**, e70067. 10.1002/DAC.70067 (2025).

[6] Naghib, A., Jafari, N. N., Hosseinzadeh, M. & Sharifi, A. A comprehensive and systematic literature review on the big data management techniques in the internet of things. *Wirel. Netw.* **29**(3), 1085–1144. 10.1007/S11276-022-03177-5 (2022).

[7] Pichumani, S., Sundararajan, T. V. P. & Ramesh, S. M. Federated stochastic gradient averaging ring homomorphism based learning for secure data aggregation in WSN. *Sci. Rep.* **15**, 18590. 10.1038/s41598-025-03257-4 (2025). 40425719 10.1038/s41598-025-03257-4 PMC12116749

[8] Sujihelen, L., Jayakumar, C. & Senthilsingh, C. SEC approach for detecting node replication attacks in static wireless sensor networks. *J. Electr. Eng. Technol.* **13**(6), 2447–2455. 10.5370/JEET.2018.13.6.2447 (2018).

[9] Guitouni, Z., Ammar, N. & Machhout, M. An efficient hardware implementation of SHA-3 using 3D cellular automata for secure wireless sensor networks. *Int. J. Inf. Secur.* **24**(3), 116–130. 10.1007/s10207-025-01007-1 (2025).

[10] Chowdhury, M., Ray, B., Chowdhury, S. & Sutharshan, R. A novel insider attack and machine learning based detection for the internet of things. *ACM Trans. Internet Things* **2**(4), 1–23. 10.1145/3466721 (2021).

[11] Feroz Khan, A. B. An enhanced multi attribute based trusted attack resistance (EMBTR) for the secure routing of sensor nodes in wireless sensor network. *Wirel. Pers. Commun.* **137**(4), 2397–2407. 10.1007/S11277-024-11504-6 (2024).

[12] Affane, A. R. & Satori, H. Machine learning attack detection based-on stochastic classifier methods for enhancing of routing security in wireless sensor networks. *Ad Hoc Netw.* **163**, 103581. 10.1016/J.ADHOC.2024.103581 (2024).

[13] Sivakumar, S., Theresa, M., Sudha, K. & Sangeethalakshmi, K. Secure wireless sensor networks: A weighted K-NN and RNN-based approach for attack detection and localization. *IETE J. Res.* **71**(3), 864–875. 10.1080/03772063.2024.2436970 (2025).

[14] Wang, H., Lu, R., Peng, Z. & Li, M. Clock synchronization with partial timestamp information for wireless sensor networks. *Signal Process.* **209**, 109036. 10.1016/j.sigpro.2023.109036 (2023).

[15] Wang, C. & Zhang, Z. Energy-efficient time synchronisation based on odd-even periodic data transmission and acknowledgement in wireless sensor networks. *Int. J. Sens. Netw.* **38**(2), 113–121. 10.1504/IJSNET.2022.121164 (2022).

[16] Li, L. et al. A secure random key distribution scheme against node replication attacks in industrial wireless sensor systems. *IEEE Trans. Ind. Inform.* **16**(3), 2091–2101. 10.1109/TII.2019.2927296 (2020).

[17] Parno B, Perrig A, Gligor V Distributed detection of node replication attacks in sensor networks. *Proceedings of the 2005 IEEE Symposium on Security and Privacy (S&P’05)*, *IEEE* 49–63. 10.1109/SP.2005.8 (2005)

[18] Xing K, Liu F, Cheng X, Du D H C Real-time detection of clone attacks in wireless sensor networks. *The 28th International Conference on Distributed Computing Systems (ICDCS ‘08)*. *IEEE* 3–10. 10.1109/ICDCS.2008.55 (2008)

[19] Anitha, S., Jayanthi, P. & Thangarajan, R. Detection of replica node attack based on exponential moving average model in wireless sensor networks. *Wirel. Pers. Commun.* **115**(2), 1651–1666. 10.1007/s11277-020-07648-w (2020).

[20] Mohindru, V., Singh, Y. & Bhatt, R. Securing wireless sensor networks from node clone attack: a lightweight message authentication algorithm. *Int. J. Inf. Comput. Secur.* **12**(2/3), 217–233. 10.1016/J.JNCA.2021.103118 (2020).

[21] Zhu B, Setia S, Krishna A V G, Jajodia S, Roy S Efficient distributed detection of node replication attacks in sensor networks. *Proceedings of the 23rd Annual Computer Security Applications Conference (ACSAC ‘07)**IEEE,* 257–267. 10.1109/ACSAC.2007.26. (2007)

[22] Li, Z. & Gong, G. On the node clone detection in wireless sensor networks. *IEEE ACM Trans. Netw.* **21**(6), 1799–1811. 10.1109/TNET.2012.2233750 (2013).

[23] Devi, P. P. & Jaison, B. Protection on wireless sensor network from clone attack using the SDN-enabled hybrid clone node detection mechanisms. *Comput. Commun.* **152**, 316–322. 10.1016/j.comcom.2020.01.064 (2020).

[24] Vaishnavi, S. & Sethukarasi, T. Detection and avoidance of clone attack in IoT based smart health application. *Intell. Autom. Soft Comput.* **31**(3), 1919–1937. 10.32604/IASC.2022.021006 (2022).

[25] Zhou, H. et al. Intrusion detection method for node replication attack based on clustering. *Transd. Microsyst. Technol.* **33**(5), 129–132. 10.13873/j.1000-97872014.05.029 (2014).

[26] Cheng, J. Research on clustered replicated node detection protocol based on Bloom filter. *Wirel. Internet Sci. Technol.* **20**(5), 14–16. 10.3969/j.issn.1672-6944.2023.05.005 (2023).

[27] Huynh, T. T. B. et al. A heuristic node placement strategy for extending network lifetime and ensuring target coverage in mobile wireless sensor networks. *Evol. Intell.* **17**(5), 3151–3168. 10.1007/s12065-024-00916-9 (2024).

[28] Anthoniraj, J. & Abdul Razak, T. CBCD: Cluster based clone detection in mobile wireless sensor networks. *Ind. J. Sci. Technol.* **9**(31), 1–10. 10.17485/ijst/2016/v9i31/86364 (2016).

[29] Sajitha, M., Kavitha, D. & Chenna Reddy, P. An optimized whale based replication node prediction in wireless sensor network. *Wirel. Netw.* **28**(4), 1587–1603. 10.1007/s11276-022-02928-8 (2022).

[30] Sujihelen, L. et al. Node replication attack detection in distributed wireless sensor networks. *Wirel. Commun. Mob. Comput.* **2022**, 7252791. 10.1155/2022/7252791 (2022).

[31] Chang, B., Zhang, X. & Bian, H. An accurate cooperative localization algorithm based on RSS model and error correction in wireless sensor networks. *Electronics* **13**(11), 2131. 10.3390/electronics13112131 (2024).

[32] Santosh Kumar, K. et al. RSSI-based optimization of static and mobile node combinations for dynamic node localization in wireless sensor networks. *Telecommun. Syst.* **87**(1), 137–149. 10.1007/S11235-024-01183-W (2024).

[33] Singh, A. & Rathee, G. FATE: Flexible attribute-based traceable encrypted data sharing scheme using smart contracts in wireless medical sensor networks. *Ann. Telecommun.* **80**(3), 323–339. 10.1007/S12243-024-01038-0 (2025).

[34] Selvi, M., Santhosh Kumar, S. V. N., Thangaramya, K. & Abdul Gaffar, H. Energy efficient trust aware secure routing algorithm with attribute based encryption for wireless sensor networks. *Sci. Rep.* **15**, 19724. 10.1038/s41598-025-03558-8 (2025). 40473671 10.1038/s41598-025-03558-8 PMC12141722

[35] Zhou, H. et al. Data reduction for black-box adversarial attacks against deep neural networks based on side-channel attacks. *Comput Secur.* **153**, 104401. 10.1016/j.cose.2025.10440 (2025).

[36] Gupta, P., Wever, M. & Hüllermeier, E. Information leakage detection through approximate Bayes-optimal prediction. *Inf. Sci.* **719**, 122419. 10.1016/j.ins.2025.122419 (2025).

[37] Kumar, S. & Singh, M. Auto-localization algorithm for mobile sensor nodes in wireless sensor networks. *J. Supercomput.* **80**(9), 13141–13175. 10.1007/s11227-024-05920-5 (2024).

[38] Raveendranadh, B. & Tamilselvan, S. An accurate attack detection framework based on exponential polynomial kernel-centered deep neural networks in the wireless sensor network. *Trans. Emerg. Telecommun. Technol.* **34**(3), e4726. 10.1002/ett.4726 (2023).

[39] Meng, Y. On a certain quadratic character sums of ternary symmetry polynomials mod p. *J. Math.* **2021**, 5572835. 10.1155/2021/5572835 (2021).

[40] Praveen, K. V., Mary, J. P. P., Ramshankar, N. & Murugesan, S. Energy-efficient communication using auto-associative polynomial convolutional neural network in WSN. *Int. J. Commun. Syst.* **38**(4), e6075. 10.1002/dac.6075 (2025).

[41] Tasic, I., Villafranca, A. & Cano, M. D. Reinforcing cybersecurity with Bloom filters: a novel approach to password cracking efficiency. *EURASIP J. Inf. Secur.* **2024**, 35–44. 10.1186/S13635-024-00183-2 (2024).

[42] Baird, I., Ghaleb, B., Wadhaj, I., Russell, G. & Buchanan, W. J. Securing IoT: Mitigating sybil flood attacks with bloom filters and hash chains. *Electronics* **13**(17), 3467. 10.3390/ELECTRONICS13173467 (2024).

[43] Huo, X. A light-weight authentication scheme in the Internet of Things using the enhanced Bloom filter. *Int. J. Adv. Comput. Sci. Appl.* **14**(1), 494–501. 10.14569/IJACSA.2023.0140154 (2023).

[44] Jin, Y. & Miyaji, A. Compact and efficient constant-time GCD and modular inversion with short-iteration. *IEICE Trans Inf. Syst.* **e106**(D9), 1397–1406. 10.1587/transinf.2022ICP0009 (2023).

[45] Gunasekaran, A. Internet of things-based wireless sensor networks for monitoring access constraint security measures in liable data aggregation. *Int. J. Commun. Syst.* **36**(17), e5596. 10.1002/dac.5596 (2023).

